# Temporomandibular disorder: otologic implications and its relationship to sleep bruxism^☆^

**DOI:** 10.1016/j.bjorl.2017.07.010

**Published:** 2017-08-23

**Authors:** Bruno Gama Magalhães, Jaciel Leandro de Melo Freitas, André Cavalcanti da Silva Barbosa, Maria Cecília Scheidegger Neves Gueiros, Simone Guimarães Farias Gomes, Aronita Rosenblatt, Arnaldo de França Caldas Júnior

**Affiliations:** a Universidade Federal de Pernambuco (UFPE), Programa de Pós-graduação em Odontologia, Recife, PE, Brazil; b Universidade Federal de Pernambuco (UFPE), Odontologia, Recife, PE, Brazil; c Universidade Federal de Pernambuco (UFPE), Recife, PE, Brazil

**Keywords:** Bruxism, Temporomandibular disorders, Otologic symptoms, Bruxismo, Disfunções temporomandibulares, Sintomas otológicos

## Abstract

**Introduction:**

Temporomandibular disorder is an umbrella term for various clinical problems affecting the muscles of mastication, temporomandibular joint and associated structures. This disorder has a multifactor etiology, with oral parafunctional habits considered an important co-factor. Among such habits, sleep bruxism is considered a causal agent involved in the initiation and/or perpetuation of temporomandibular disorder. That condition can result in pain otologic symptoms.

**Objective:**

The aim of the present study was to investigate the relationship between temporomandibular disorder and both otologic symptoms and bruxism.

**Methods:**

A total of 776 individuals aged 15 years or older from urban areas in the city of Recife (Brazil) registered at Family Health Units were examined. The diagnosis of temporomandibular disorder was determined using Axis I of the Research Diagnostic Criteria for temporomandibular disorders, addressing questions concerning myofascial pain and joint problems (disk displacement, arthralgia, osteoarthritis and osteoarthrosis). Four examiners had previously undergone training and calibration exercises for the administration of the instrument. Intra-examiner and inter-examiner agreement was determined using the Kappa statistic. Individuals with a diagnosis of at least one of these conditions were classified as having temporomandibular disorder. The diagnosis of otologic symptoms and bruxism was defined using the same instrument and a clinical exam.

**Results:**

Among the individuals with temporomandibular disorder, 58.2% had at least one otologic symptom and 52% exhibited bruxism. Statistically significant associations were found between the disorder and both otologic symptoms and bruxism (*p* < 0.01 for both conditions; OR = 2.12 and 2.3 respectively). Otologic symptoms and bruxism maintained statistical significance in the binary logistic regression analysis, which demonstrated a 1.7 fold and twofold greater chance of such individuals have temporomandibular disorder, respectively.

**Conclusion:**

The logistic regression analysis demonstrated strong associations between the disorder and both otologic symptoms and bruxism when analyzed simultaneously, independently of patient age and gender.

## Resumo

**Introdução:**

A Disfunção Temporomandibular é um termo que engloba vários problemas clínicos que afetam os músculos da mastigação, a articulação temporomandibular e estruturas associadas. Este distúrbio tem uma etiologia multifatorial, com hábitos parafuncionais orais considerados um importante cofator. Entre esses hábitos, o bruxismo do sono é considerado um agente causador envolvido no desenvolvimento e/ou perpetuação de disfunção temporomandibular. Esse problema pode resultar em sintomas otológicos dolorosos.

**Objetivo:**

Investigar a relação entre disfunção temporomandibular e os sintomas otológicos e bruxismo.

**Método:**

Foram examinados 776 indivíduos com idade igual ou superior a 15 anos de áreas urbanas da cidade de Recife (Brasil) registrados nas Unidades de Saúde da Família. O diagnóstico da disfunção foi determinado utilizando o Eixo I dos Critérios de Diagnóstico de Pesquisa para Distúrbios Temporomandibulares, abordando questões relativas a dor miofascial e problemas articulares (luxação discal, artralgia, osteoartrite e osteoartrose). Quatro examinadores foram treinados para a administração do instrumento. A concordância intraexaminador e interexaminador foi determinada usando a estatística Kappa. Os indivíduos com diagnóstico de pelo menos uma dessas condições foram classificados como tendo disfunção temporomandibular. O diagnóstico de sintomas otológicos e bruxismo foi definido utilizando o mesmo instrumento de diagnóstico e exame clínico.

**Resultados:**

Entre os indivíduos com a disfunção, 58,2% apresentaram pelo menos um sintoma otológico e 52% apresentaram bruxismo. Foram encontradas associações estatisticamente significativas entre a disfunção temporomandibular e ambos os sintomas otológicos e bruxismo (*p* < 0,01 para ambos os problemas, OR = 2,12 e 2,3, respectivamente). Os sintomas otológicos e o bruxismo mantiveram significância estatística na análise de regressão logística binária, o que demonstrou uma probabilidade de 1,7 e 2 vezes maior chance de que esses indivíduos tenham disfunção temporomandibular, respectivamente.

**Conclusão:**

A análise de regressão logística demonstrou associações fortes entre disfunção temporomandibular e sintomas otológicos e bruxismo quando analisados simultaneamente, independentemente da idade e sexo do paciente.

## Introduction

Temporomandibular disorder (TMD) is an umbrella term for various clinical problems affecting the muscles of mastication, temporomandibular joint (TMJ) and associated structures.^1^ This disorder has a multifactor etiology,^2^ with oral parafunctional habits considered an important co-factor.^3^ Among such habits, sleep bruxism is considered a causal agent involved in the initiation and/or perpetuation of TMD.^4^ Bruxism is the act of grinding or clenching the teeth in a parafunctional or unconscious manner. Bruxism causes hyperactivity of the muscles of mastication due to non-functional mandibular movements, which can result in pain symptoms and is therefore an important contributing factor to changes in the TMJ.^5^

The clinical symptoms of TMD include clicking or crackling sounds in the TMJ, difficulty opening and closing the mouth and pain, the latter of which can spread to different regions of the head, including the pre-auricular and auricular regions.^5^ A number of studies have suggested an association between otologic symptoms and TMD.^6^, ^7^, ^8^ The symptoms most frequently reported in the literature are ringing in the ears, otalgia, a sensation of ear fullness, loss of hearing and dizziness.^1^, ^6^, ^7^ The association of these symptoms with TMD is thought to be multi-causal, due to anatomic, neurologic and emotional relationships.^9^

In this context, there is a likely hypothesis that hyperactivity of the muscles of mastication may contract the tensor tympani muscle and tympanic membrane, which would result in auditory tube dysfunction, with symptoms of a sensation of ear fullness, loss of balance and hearing loss.^2^ Due to the scarcity of studies in the literature, the aim of the present study was to investigate the association between TMD and both otologic symptoms and sleep bruxism.

## Methods

A cross-sectional study was conducted with a sample of 776 individuals aged 15 years or older from urban areas registered at Family Health Units. No restrictions were imposed regarding gender or ethnicity. Age was categorized based on an adaptation of the criteria of the World Health Organization: 15–18, 19–24, 25–44, 45–59 and 60 years or older.^10^

Multi-stage sampling was used to determine the sample size and obtain a representative sample of the entire city. First, systematic sampling was employed to define the neighborhoods in the health districts that would take part in the study. Systematic sampling was then performed to select the Primary Health Units, from which 776 volunteers at their respective health units were randomly selected.

The whole project including the both informed consent received approval from the Human Research Ethics Committee (n° 05650512.9.0000.5208). All participants signed a statement of informed consent and those diagnosed with TMD were referred to a reference center for treatment. For those under 18 years of age, an informed consent to perform the examinations came from the parents or guardians properly signed. This research has been conducted in full accordance with the World Medical Association Declaration of Helsinki.^11^

The diagnosis of TMD was determined using Axis I of the Research Diagnostic Criteria for temporomandibular disorders (RDC/TMD), addressing questions concerning myofascial pain and joint problems (disk displacement, arthralgia, osteoarthritis and osteoarthrosis). The diagnosis of myofascial pain is based on the report of pain in the temples, Jaw, face, preauricular area or inside the ear at rest or during function. Pain reported by the individual in response to palpation of three or more muscle groups. There is also the presence of muscular pain with or without limitation of oral opening. Disk displacement is diagnosed by opening, closing, lateral excursion, or protruding Jaw movements; reciprocal clicks on the TMJ (disk displacement with reduction), opening limitation (disk displacement without reduction), or disk displacements without reduction and without signs of limiting mouth opening. Arthralgia is detected through the presence of one or more pain symptoms in the joint region, pain in the joint during the maximal opening without aid, pain in the joint during the opening with aid, pain in the joint during the lateral excursion if gross crackling. TMJ osteoarthritis when arthralgia and coarse crackling and TMJ osteoarthrosis were observed when there was no evidence of arthralgia and with coarse crackling (Manfredini eixo 1).^12^ Individuals with a diagnosis of at least one of these conditions were classified as having TMD. Four examiners had previously undergone training and calibration exercises for the administration of the RDC/TMD. Intra-examiner and inter-examiner agreement was determined using the Kappa statistic (*K* = 0.90 and 0.82, respectively).

The presence of otologic symptoms was considered when at least one of the following was present: tinnitus, otalgia, ear fullness, dizziness and hearing loss, which were all diagnosed by patients’ report. Sleep bruxism was diagnosed by self-report or report of a family member about grinding or clenching during sleep using RDC/TMD (Axis II). Axis II consists of a questionnaire with 31 items, divided into socio-demographic, socioeconomic, psychological (sub-scales of depression and non-specific physical symptoms – painful and non-painful), psychosocial (degree of severity of chronic pain – intensity of pain and related disability); Signs and symptoms related to the patient and the limitation scale in mandibular function (limitations related to mandibular functioning). The mean score is calculated by summing the score of individual items, allowing patients to be assessed within normal, moderate or severe levels of depression, and specific and non-specific physical symptom scales. However, for the purpose of this article only related relevant information on sleep bruxism and otological symptoms was extracted.^13^, ^14^

It must be emphasized that all patients with otologic symptoms did not know about their diagnosis on temporomandibular dysfunction, so they were not on treatment. Furthermore, there are not treatment for TMD at the public health centers in the state of Pernambuco. All patients properly diagnosed are referred to state dental colleges.

Individuals with neurological disorders, those with a history of tumor in the head and neck region, those who made continuous use or for at least the previous three days of anti-inflammatory agents, analgesic or corticoids, those unable to understand or respond to the RDC/TMD, those who reported a history of rheumatic disease, those with pain of an odontogenic origin and those with a primary otalgia were excluded from the study. This information was extracted from the patient charts at the health services.

For the statistical analysis, the Statistical Package for the Social Sciences (SPSS, version 20.0) was employed for the data entry and statistical calculations. The Shapiro–Wilk was used to determine the distribution of the data (normal or non-normal). Pearson's chi-square test was used to test associations between the dependent and independent variables. A 5% margin of error with a 95% significance level (*p* < 0.05) was considered for all analyses. A regression model was created to identify possible confounding variables and explanatory variables.

## Results

A total of 776 individuals aged 15–85 years (mean: 39.88 years; median: 39 years) participated in the present study. The prevalence of TMD in the sample analyzed was 35.4%. Among the 275 individuals diagnosed with TMD, 88.4% were female. The prevalence was greater in the age group of 45–59 years, corresponding to 41% of the affected individuals (Table 1).

**Table 1:** Distribution of participants with TMD according to otologic symptoms and bruxism.

Variables	TMD			
	Yes *n* (%)	No *n* (%)	Total (%)	Odds (95% CI)
*Gender (p* *=* *0.029)*				1.6 (1.04–2.49)
Male	243 (37)	413 (63)	656 (100)	
Female	32 (26.7)	88 (73.3)	120 (100)	

*Age (p* *=* *0.32)*				–
15–18 years	10 (25.6)	29 (74.4)	39 (100)	
19–24 years	29 (33.3)	58 (66.7)	87 (100)	
25–44 years	132 (36.5)	230 (63.5)	362 (100)	
45–59 years	87 (41)	125 (59)	212 (100)	
>59 years	17 (22.4)	59 (77.6)	76 (100)	

*Otologic symptoms (p* *=* *0.001)*				2.1 (1.57–2.87)
Yes	160 (44.7)	198 (55.3)	358 (100)	
No	115 (27.5)	303 (72.5)	418 (100)	

*Sleep bruxism (p* *=* *0.001)*				2.2 (1.70–3.12)
Yes	143 (47.2)	160 (52.8)	303 (100)	
No	132 (27.9)	341 (72.1)	473 (100)	

Among the individuals with TMD, 58.2% had at least one otologic symptom and 52% exhibited sleep bruxism. Pearson's Chi-square test demonstrated statistically significant associations between TMD and both otologic symptoms and sleep bruxism (*p* < 0.01 for both conditions), with an odds ratio of 2.12 for otologic symptoms and 2.3 for sleep bruxism.

Binary logistic regression analysis was performed to evaluate the behavior of the covariables simultaneously with the outcome (TMD). Otologic symptoms and sleep bruxism maintained statistical significance, demonstrating a 1.7 fold and twofold greater chance of such individuals developing TMD, respectively (Table 2).

**Table 2:** Final logistic regression model for TMD according to gender, age, otologic symptoms and bruxism.

	*B*	S.E.	Wald	df	*p*-value	OR	95% CI for OR	
							Lower limit	Upper limit
Gender	0.425	0.229	3.453	1	0.063	1.530	0.977	2.395
Age	0.013	0.082	0.026	1	0.871	1.013	0.863	1.190
Otologic symptoms	0.556	0.160	12.073	1	0.001	1.744	1.274	2.386
Sleep bruxism	0.702	0.160	19.145	1	0.000	2.018	1.473	2.764
Constant	−1.882	0.488	14.862	1	0.000	0.152		
Goodness of fit^a^	0.394							

a Determined using Hosmer–Lemeshow test.

## Discussion

The aim of the present study was to investigate comorbidities that may be associated with TMD. The literature reports associations between this disorder and parafunctional habits.^15^, ^16^ The studies cited indicated that parafunctional habits can alter the harmony of the stomatognathic system and are therefore considered a significant element in the etiology and progression of muscle and TMJ disorders. Among such habits, the present study found that sleep bruxism was associated with TMD, which is in agreement with findings described in previous studies.^15^, ^16^

It is important to note that sleep related movement disorders has also been described in other instruments as in the International Classification of Sleep Disorders (ICSD-3), recognized as an important clinical text for the diagnosis of sleep disorders classifying sleep disorders into six major categories (Insomnia, Sleep Related Breathing Disorders, Central Disorders of Hypersomnolence, Circadian Rhythm Sleep-Wake Disorders, Parasomnias and Sleep Related Movement Disorders). However, the approach of the present study focused on the use of RDC considering its relevance for epidemiological purposes.^17^ Bruxism can lead to muscle hyperactivity, resulting in pain in the muscles of mastication. In the presence of pain and other symptoms, the stomatognathic system may perform compensations to allow chewing, speaking and swallowing with efficiency and comfort. However, this can have a negative effect in the long term; as such compensations are not necessarily healthy and may contribute to the progression of TMD. Moreover, the indiscriminant use of analgesics without medical supervision, as often occurs in individuals with TMD, can mask symptoms and lead to an aggravation of the problem.^18^

The analysis of the sample also indicated a positive association between otologic symptoms and TMD, which is in agreement with some reports found in the literature.^19^, ^20^ A previous study found that signs of TMD are predictors of the development of some otologic symptoms, such as ringing in the ears.^21^ However, the relationship between TMD and otologic symptoms had not yet been fully clarified. Nonetheless, this relationship has been grounded on embryological, anatomic and functional relationships in the region that encompasses the TMJ, muscles innervated by the trigeminal nerve and structures of the middle ear.

Among other factors, it has been suggested that muscle changes in individuals with TMD, such as spasms in the lateral pterygoid muscle, cause hypertonia of the tensor tympani muscle, thereby generating changes in the auditory tube and a consequent reduction in the ventilation of the middle ear.^19^ Thus, the abnormal activity of the tensor tympani muscle is associated with otologic symptoms, such as a sensation of ear fullness, tinnitus, dizziness and hypo/hyperacusis, without the existence of another illness of an otologic nature.^2^

A study conducted by Felício et al.^18^ suggest that the abnormal solicitation of the muscles of mastication due to bruxism not only cause pain, but can contribute to changes in the TMJ, thereby triggering TMD. Moreover, as stated above, hyperactivity of the muscles of mastication can result in abnormal activity of the tensor tympani muscle, leading to otologic symptoms.^18^

It is also important to consider cultural, environmental and biological factors that may play an important role in the maintenance and progression of TMD.^22^, ^23^, ^24^ A greater prevalence rate of TMD was found among females in the present study. According to Poveda Roda et al., TMD is four times more frequent in women, who also tend to seek treatment three times more than men.^25^ Studies have suggested that estrogen receptors modulate metabolic functions with regard to the relaxation of ligaments, which may contribute to the progression of TMD.^25^ In this context, it should be stressed that the most affected age group corresponds to important hormonal changes, such as menopause, which suggests participation in this process, according to previous studies.^26^, ^27^ However, both gender and age lost their statistical significance in the logistic regression analysis, which demonstrated strong associations between TMD and both otologic symptoms and sleep bruxism when analyzed simultaneously, independently of the age and gender of the patients. Thus, it is important to consider the TMD-sleep bruxism-otologic symptoms triad in clinical and epidemiological diagnostic systems so that patients can be correctly diagnosed and treated.

## Conclusion

The logistic regression analysis demonstrated strong associations between TMD and both otologic symptoms and bruxism when analyzed simultaneously, independently of patient age and gender.

## Conflicts of interest

The authors declare no conflicts of interest.
